# The Genetic Basis of a Rare Flower Color Polymorphism in *Mimulus lewisii* Provides Insight into the Repeatability of Evolution

**DOI:** 10.1371/journal.pone.0081173

**Published:** 2013-12-03

**Authors:** Carrie A. Wu, Matthew A. Streisfeld, Laura I. Nutter, Kaitlyn A. Cross

**Affiliations:** 1 Department of Biology, University of Richmond, Richmond, Virginia, United States of America; 2 Institute of Ecology and Evolution, University of Oregon, Eugene, Oregon, United States of America; Michigan State University, United States of America

## Abstract

A long-standing question in evolutionary biology asks whether the genetic changes contributing to phenotypic evolution are predictable. Here, we identify a genetic change associated with segregating variation in flower color within a population of *Mimulus lewisii*. To determine whether these types of changes are predictable, we combined this information with data from other species to investigate whether the spectrum of mutations affecting flower color transitions differs based on the evolutionary time-scale since divergence. We used classic genetic techniques, along with gene expression and population genetic approaches, to identify the putative, loss-of-function mutation that generates rare, white flowers instead of the common, pink color in *M. lewisii*. We found that a frameshift mutation in an anthocyanin pathway gene is responsible for the white-flowered polymorphism found in this population of *M. lewisii*. Comparison of our results with data from other species reveals a broader spectrum of flower color mutations segregating within populations relative to those that fix between populations. These results suggest that the genetic basis of fixed differences in flower color may be predictable, but that for segregating variation is not.

## Introduction

The past several years have seen an explosion of experimental studies seeking to identify the genetic and molecular bases of adaptive traits [1]–[4]. An important question that emerges from these studies is whether the genetic changes contributing to phenotypic evolution are predictable. In other words, does natural selection preferentially target specific genes – or certain types of mutations – during adaptation? This question has been presented and discussed in many forms over the past several years, with the recognition that a synthesis of developmental biology, evolution, and population genetics is needed to draw firm conclusions about the evolutionary processes influencing the genetics of adaptive traits [5]–[11].

One idea that has been proposed to explain the preferential use of particular types of mutations suggests that the evolutionary time-scale since divergence may shape patterns of predictability [9], [12]. Specifically, in their survey of the molecular changes contributing to phenotypic evolution in plants and animals, Stern and Orgogozo [9], [12] observed that *cis*-regulatory changes dominated transitions in morphology between species (what they referred to as ‘long-term’ evolution), but phenotypic variation within species (i.e. ‘short-term’ evolution) was caused by an elevated frequency of null coding mutations. To explain this observation, they argued that alleles that persist between species likely have been exposed to heterogeneous environmental and selective pressures over a longer time-scale than alleles that vary within species. Because the probability of fixation of an advantageous mutation is proportional to its net selective advantage, only those mutations with the fewest negative pleiotropic effects should be favored over the long term [13], [14]. Thus, *cis*-regulatory mutations with reduced deleterious pleiotropic effects are expected to persist longer than null coding mutations.

These results suggest that substantial selective filters accompany longer evolutionary time-scales to generate non-random biases in the spectrum of mutations found between species compared to those that occur within species [15]. However, the definition of species can vary tremendously across different taxonomic treatments, and the delineation of species boundaries will depend on which taxonomy is followed. Therefore, polymorphisms that would be defined as within-species polymorphisms following one taxonomy could be considered interspecific in another. However, because populations typically fix adaptive alleles during an initial period of divergence that precedes speciation [16], the process of fixation may be directly responsible for the pattern observed by Stern and Orgogozo [9]. Therefore, we expect that variants that have fixed between populations will be exposed to similar selective filters regardless of whether those differentiated populations are considered different species or not. One way to test this hypothesis is to compare the spectrum of mutations (i.e. the substitution spectrum [10]) involved in the repeated fixation of a single trait between populations to the spectrum of mutations affecting segregating polymorphism of the same trait within populations. If the spectra differ across these alternate scales, this suggests that among the set of possible mutations that arise within populations to confer the phenotype, only some of them are fixed during evolution.

Transitions in flower color provide an excellent opportunity to address whether fixed differences between populations have a different genetic basis than those that segregate within populations. Numerous evolutionary transitions in flower color occur between populations and species [17], and segregating polymorphisms within populations are also common [18]. Moreover, the biochemical pathways and regulatory networks that control the production of floral pigments, particularly those that determine anthocyanin pigmentation, are well studied and highly conserved [19], [20]. Because of their prevalence in nature, we focus here on the genetic basis for changes in floral anthocyanin pigment intensity within and between populations, which are often determined by variation in the concentration of pigment deposited in the flower.

**Figure 1:**
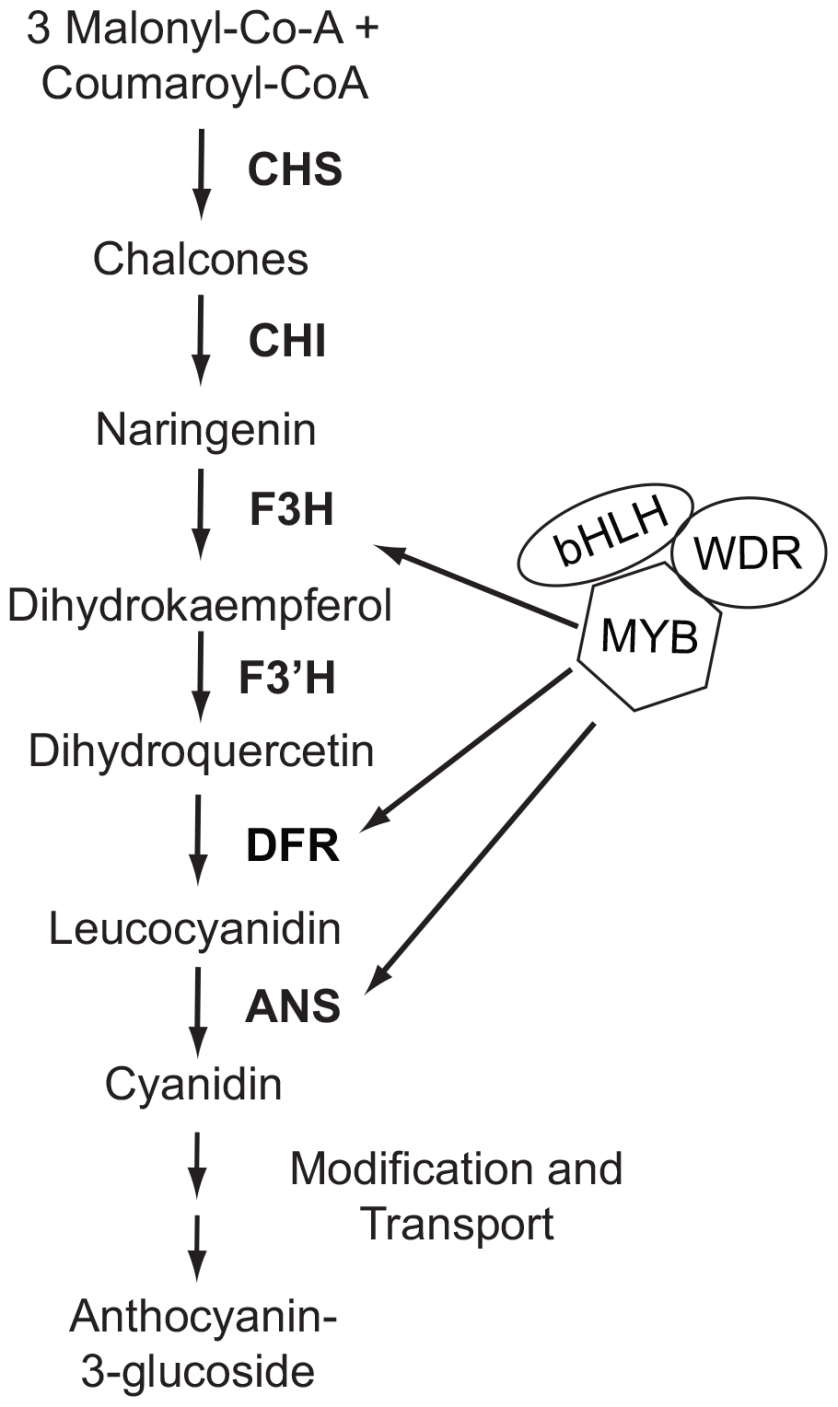
Schematic of the anthocyanin biosynthetic pathway leading to the production of cyanidin in *Mimulus lewisii*. Transcriptional activation of the enzyme-coding genes by the transcription factor complex is indicated by arrows and reflects the pattern observed in other *Mimulus* species [29], [30]. Abbreviations: CHS, chalcone synthase; CHI, chalcone isomerase; F3H, flavonol 3-hydroxylase; F3′H, flavonoid 3′-hydroxylase; DFR, dihydroflavonol 4-reductase; ANS, anthocyaninidin synthase. R2R3-MYB (MYB), basic helix-loop-helix (bHLH), and WD-40 repeat (WDR) transcription factors regulate enzymatic expression.

Anthocyanin biosynthesis requires the function of at least six enzymes, many of which are coordinately regulated by three interacting transcription factor proteins [21] (Fig. 1). Therefore, to generate changes in anthocyanin pigment intensity within or between species, flux through this pathway must be altered. In principle, such alteration may be caused in any of four ways: (i) by *cis*-regulatory changes to enzyme-coding genes that directly affect enzymatic expression; (ii) by coding-sequence mutations in enzyme-coding genes; (iii) by *cis*-regulatory changes to anthocyanin-regulating transcription factors; or (iv) by coding-sequence mutations to anthocyanin-regulating transcription factors [10]. Despite these possibilities, the repeated evolution of anthocyanin intensity changes that affect flower color transitions between populations or species appears to follow a strong pattern of predictability, with all known cases resulting from the fixation of mutations in one class of pigment-regulating transcription factor [10].

In the current study, we explore the genetic changes associated with segregating variation in floral pigment intensity within populations. Across its range, the perennial wildflower *Mimulus lewisii* (Phrymaceae) synthesizes anthocyanin pigments in its vegetative and floral tissues, resulting in flowers that display a constitutively dark-pink color and leaves that turn purple upon stress. However, occasional reports suggest that rare, white-flowered variants exist within natural populations. Here, we characterize the genetic basis of one of these white-flowered alleles that segregates at low frequency in a population in Oregon. We take advantage of the wealth of information available for the anthocyanin pathway and use classic genetic techniques, along with gene expression and population genetic approaches to identify the putative, loss-of-function mutation that generates white flowers. We then combine this information with data on the genetic basis of flower color shifts that segregate within populations in other species to examine whether the spectrum of mutations segregating in populations differs from the spectrum of mutations that fixes between populations.

## Materials and Methods

### Study system

*Mimulus lewisii* Pursh (Phrymaceae) is a widely-distributed, self-compatible, perennial herb that grows along stream banks in western North America [22], [23]. Primarily bee-pollinated, *M. lewisii* plants typically have pink flowers with two yellow ridges of hairs that are presumed to be nectar guides [24]. The species is comprised of two well-supported sister clades, a southern race with populations in the Sierra Nevada Mountains of California, and a northern race distributed from southern coastal Alaska through the Cascade Mountains of southern Oregon [25]. Rarely, populations of *M. lewisii* containing white-flowered individuals have been reported in the Pacific Northwest.

**Figure 2:**
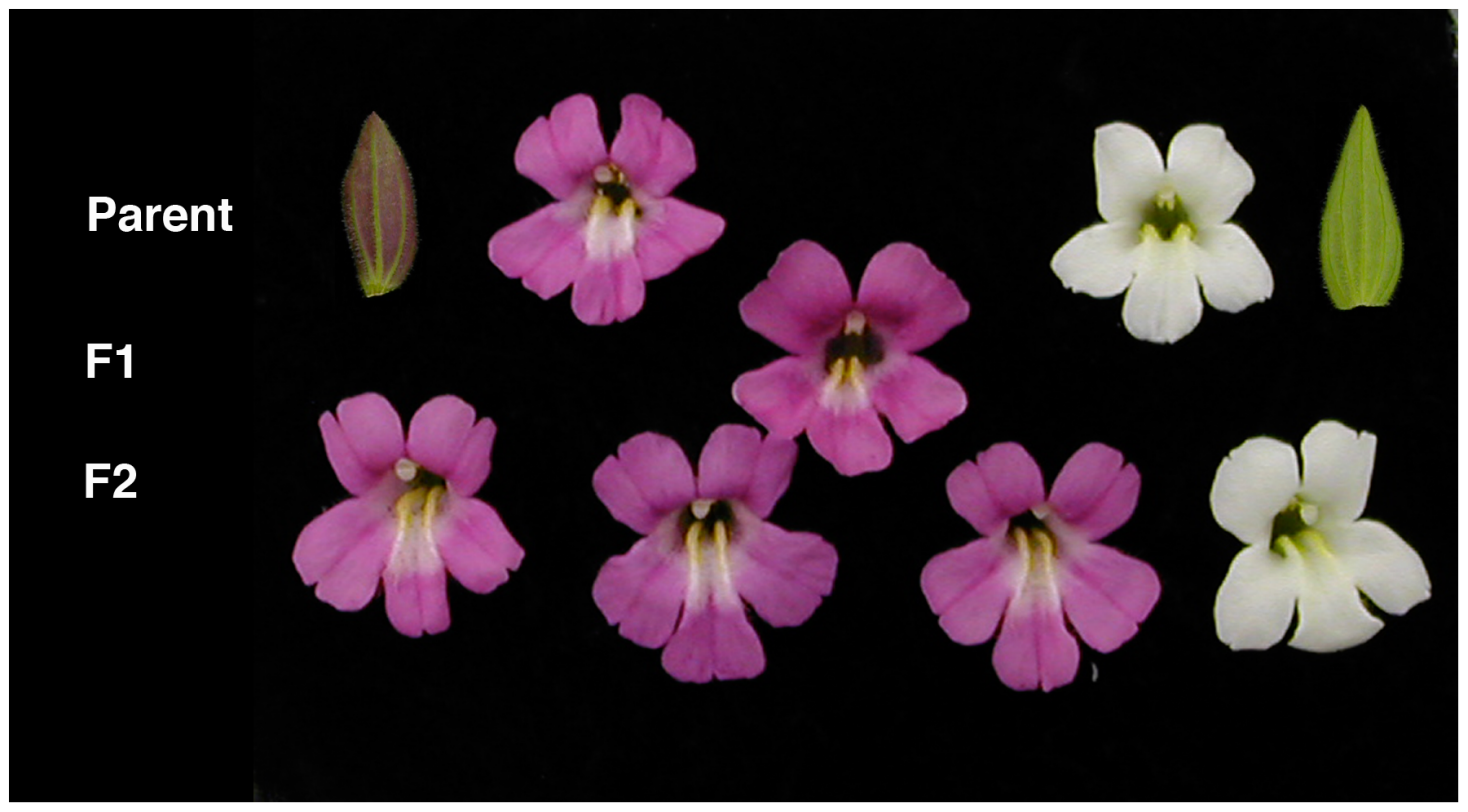
Pigmentation phenotypes of *Mimulus lewisii* from Crater Lake, Oregon. Top row: Leaf and flower from a typical wild-type individual (left) show dark pink coloration, while the rare, white-flowered morph does not show evidence for anthocyanin production in either flower or leaf tissue. Middle and bottom rows: F_1_ hybrid crosses show that the wild type, pink flower is dominant to white, and segregation ratios in the F_2_ generation do not differ from 3∶1, suggesting a recessive allele at a single locus is responsible for white flowers.

We located a population of *M. lewisii* near Crater Lake in the Oregon Cascades (CRA; N 42°53.062′ and W 122°05.952′) that contained rare, white-flowered individuals segregating within an otherwise pink-flowered population (Fig. 2). We collected rhizomes from the two white-flowered individuals that were observed in bloom within this population in August 2006 (permission granted by the US National Park Service, permit CRLA-2006-SCI-0002). Rhizomes were transported back to the Duke University greenhouses, where they were planted in moist Fafard 4P potting mix and maintained in 16 h days. Upon flowering, individuals were self-fertilized and seeds collected. Flower buds from four pink-flowered plants from this population were collected in RNA-Later and transported back to the lab, where the samples were stored at -80°C. In addition, seeds and/or flower buds were obtained from representative pink-flowered *M. lewisii* plants from populations in the south-central Oregon portion of the Modoc Plateau (MPL; provided by Paul Beardsley) and Stevens Point, Washington (UW2; provided by H. D. Bradshaw, Jr.). This allopatric MPL population was chosen as the source of wild-type pink-flowered *M. lewisii* for our study, rather than the population at Crater Lake, to minimize the possibility that the pink-flowered individuals used for comparison were derived from natural cross pollination with a white-flowered individual. Although we cannot know for sure whether any white alleles are segregating at extremely low frequency in this MPL population, extensive sampling notes (P. Beardsley, personal communication), herbarium records, field guides, and other sources have failed to report white-flowered individuals in MPL or nearby populations.

### Morph-specific pigment production

Previous work in *M. lewisii* has shown that the pink color of wild-type flowers results primarily from the anthocyanidin pigment cyanidin [26]. To determine whether the level of cyanidin production was reduced in the leaf and floral tissue of white-flowered individuals, we extracted anthocyanidins from 200 mg of bud and 225 mg of leaf tissue from each of three plants per color morph following previously described methods [27]. Floral anthocyanin extractions were also performed on buds from F_1_ hybrids between the color morphs (see below). Extracts were dried and re-suspended in methanol with 1% HCl (500 µl for leaf tissue, 1.5 ml for bud tissue), and 200 µl was analyzed with a BioRad SmartSpec Plus spectrophotometer. Absorbance was recorded at 540 nm, the wavelength at which peak absorbance (λ_max_) occurred for pink-flowered individuals (data not shown). Pigment production was compared with a t-test (leaf tissue) or one-way analysis of variance (floral tissue; JMP10, SAS Institute, Cary, North Carolina, USA), according to the model: *y* = μ+*T*+*E*, where *T*  =  type of plant (fixed effect) and *E*  =  residual error. Data and script for both analyses are deposited in DRYAD (http://doi.org/10.5061/dryad.d3f56).

### Segregation of flower color in experimental F2s

To determine whether flower color was a quantitative character, we conducted classic segregation analysis. Plants grown from field-collected rhizomes at Duke University were self-fertilized to generate the parental individuals. Reciprocal F_1_ seeds were generated by crossing a white-flowered individual from CRA with a pink-flowered individual from MPL in the Duke University greenhouses, and were transported to the University of Richmond for planting under similar conditions in a Percival 36-VLT chamber. Upon maturation, F_1_ individuals were self-fertilized to produce F_2_ seeds. From one such cross, 96 F_2_ offspring were grown in standard potting mix under 16 h days until flowering and scored for flower color as pink or white. We tested whether segregation patterns deviated from Mendelian ratios using a goodness-of-fit test.

### Cloning of anthocyanin pathway genes

From five white-flowered CRA and pink-flowered MPL individuals, partial coding sequences from four of the six core structural anthocyanin genes (*chalcone synthase [Chs], flavanone 3-hydroxylase [F3h], dihydroflavonol 4-reductase [Dfr]*, and *anthocyanidin synthase [Ans]*) were cloned using degenerate primers, and reverse-transcribed polymerase chain reaction (RT-PCR). Total RNA was extracted from floral buds using an RNeasy kit (Qiagen, Valencia, CA). First strand complementary DNA (cDNA) synthesis was performed using SuperScript II reverse transcriptase according to the manufacturer's specifications (Invitrogen, Carlsbad, CA). Degenerate primers were designed based on conserved regions in alignments from two closely-related *Mimulus* species (*M. guttatus* and *M. aurantiacus*). Primer sequences are presented in Table S1. PCR products were cloned into the pCR 2.1 TOPO vector (Invitrogen), and three to five clones for each gene were sequenced using primers M13F and M13R. While the anthocyanin pathway genes may be found in multiple copies, only single copies were identified from *M. lewisii* floral tissue. Moreover, BLAST searches against the NCBI protein database yielded the highest similarity with sequences of previously characterized anthocyanin genes from other *Mimulus* species.

### Co-segregation of anthocyanin pathway genes with flower color

As an initial screen to test if variation between the color morphs co-segregated with sequence variation at any of the structural genes, we sequenced partial coding regions for the four loci from 5 white-flowered and 5 pink-flowered F_2_s from the cross described above.

Based on these results, *MlDfr* emerged as a potential candidate to explain the flower color change in *M. lewisii* (see Results). To confirm this, we used a new set of controlled crosses between a white-flowered and pink-flowered individual to score a total of 96 F_2_ progeny for flower color (pink or white) and genotype at *MlDfr*. Genotypes were determined using PCR-RFLP from genomic DNA extracted from bud tissue (DNeasy Plant Mini Kit, Qiagen). Primers were designed to amplify a 630 bp fragment of *MlDfr*. Within this fragment, the *MlDfr* allele from the white-flowered parent contains a SNP relative to the allele from the pink-flowered parent used in this cross that introduces a recognition site for the restriction enzyme *SspI*. Following amplification, PCR products were digested with *Ssp*I for 1 hr at 37°C, as per the manufacturer's protocol (New England Biolabs (NEB), Ipswich, MA). Digested products were separated in 1.5% agarose gels containing ethidium bromide. Due to the co-dominance of this PCR-RFLP marker, heterozygous individuals were distinguishable from both homozygotes.

### Qualitative gene expression assays

In addition to coding sequence mutations in pathway enzymes, mutations that affect gene expression can also account for the differences in flower color between the morphs of *M. lewisii*.

Expression differences can occur either via *cis*-regulatory changes to pathway enzymes or via coding or *cis*-regulatory mutations in transcription factors that regulate the pathway enzymes. In all other species that have been examined, multiple anthocyanin genes have been shown to be co-regulated by a common set of transcription factors [20], [21], [28]. Moreover, cases where anthocyanin pigmentation differences are caused by altered expression levels typically involve considerable changes in transcript abundance [29]–[32]. Therefore, to examine whether prominent differences in gene expression could explain the lack of pigment production in white-flowered plants, we conducted qualitative gene expression assays. From the cloned sequences described above, *M. lewisii*-specific primers (Table S2) were designed for each of the four structural genes (*Chs, F3h, Dfr*, and *Ans*). We then performed PCR (RT-PCR) from five individuals of each flower color type. Floral bud and leaf tissue were collected separately, and RNA extracted using the RNeasy kit (Qiagen, Valencia, CA). Total RNA (500 ng) was used to make first-strand cDNA, followed by 35 cycles of PCR. Amplified PCR products were visualized on 1% agarose gels stained with ethidium bromide. The constitutively expressed gene *Ef1*α, which encodes the translation elongation factor, was included as a positive control for the presence of cDNA in the PCRs [29]. Specificity of the primers was confirmed by sequencing the PCR products.

### Cloning full-length sequence of *MlDfr*

We used two approaches to obtain full-length coding sequences of the pink and white alleles of *MlDfr*. First, we used the 3′ rapid amplification of cDNA ends (RACE) procedure (Invitrogen) according to manufacturer's specifications to clone the 3′ end of the gene, including the 3′ untranslated region. Second, to obtain the 5′ end, including the start codon and the 5′ untranslated region, we aligned the *Dfr* gene sequences from *M. guttatus* (published on Phytozome v. 8.0) and *M. aurantiacus* [29]. In a region of high sequence similarity located 162-bp 5′ of the start codon, a degenerate PCR primer was designed and used in conjunction with the degenerate PCR primers described above to amplify 1016-bp of *MlDfr* from pink and white floral cDNA. Species-specific primers designed in the 5′ and 3′ UTRs were then used to amplify the entire coding region from two white-flowered plants from CRA, three pink-flowered plants from CRA, and a pink-flowered plant from MPL and UW2 using high fidelity DNA polymerase (Bio-Rad, Hercules, CA). These samples were chosen to balance sampling depth within a population, and breadth across representative pink-flowered populations, to identify mutations that were unique to sequences from white-flowered plants. The full-length coding regions were directly sequenced from each individual.

### Allele frequency in the white-flowered population

From the above sequences, a 2-bp indel in the coding region of *MlDfr* emerged as the most likely candidate to explain the presence of white flowers in the CRA population (see Results). To provide an unbiased estimate of the frequency of this allele in CRA, we determined the genotype at this 2-bp indel from 19 individuals collected from this natural field population. Because *M. lewisii* spreads via rhizomes, we attempted to avoid sampling the same individual plant multiple times by collecting leaf samples in the field from as many distinct groups of plants as could be obtained in this relatively small population. In addition, to minimize sampling biases during collection, we sampled plants after they were finished flowering, at which point flower color could not be determined by visual inspection. Leaves were dried in silica and genomic DNA was isolated as above. PCR was performed from genomic DNA using the forward primer (5′-GACGAAGCAGTCCAAGGTTGTGCATGTGTGT-3′) that anneals 1-bp upstream of the 2-bp indel and the reverse primer (5′-TGGCTTTATCACTTCATTCTGC-3′). This forward primer contains a mismatch with the template DNA, such that the indel plus this mismatch differentially introduces a *MslI* restriction site. Following PCR, products were digested with *MslI* (NEB), as directed, and alleles were separated on 2% high resolution agarose gels (Apex), stained with ethidium bromide. PCR products were direct sequenced from a subset of individuals to validate genotype calls. No errors were detected.

## Results

### Variation in pigment production is consistent across tissues

**Figure 3:**
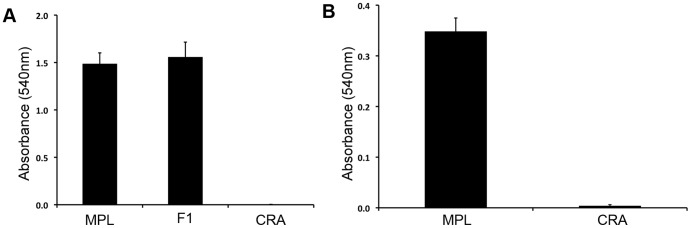
Morph-specific cyanidin production in white- and pink-flowered plants. Bars are absorbance means ± SE (*n* = 3 per genotype) for floral (A) and leaf tissue (B) pigment extractions. Genotypic classes with significant differences in mean flower color are indicated with different letters. Note the different scales for the two tissue types.

To determine the extent that anthocyanin production was reduced in white flowers relative to pink flowers, we extracted anthocyanin pigments from floral tissue of each morph. Moreover, to determine whether differences in anthocyanins between the morphs were restricted to floral tissue, we also extracted anthocyanins from leaves. Pink-flowered *M. lewisii* produced cyanidin in its leaves and flowers, but pigments were absent from both leaf and floral tissues of white-flowered plants (Figs. 2, 3). These differences were statistically significant (leaf tissue: t = 7.397, df = 2.08, *P* = 0.0085; floral tissue: ANOVA, F_2,6_ = 19.85, *P* = 0.0023, Tukey's HSD for white vs. pink parental plants, *P* = 0.0043), and suggest that a loss-of-function mutation that disrupts anthocyanin production in multiple tissues is responsible for white flowers.

### White flowers are due to a recessive allele at a single locus

To explore the genetic architecture of this difference in flower color, we conducted classic genetic crosses between white-flowered and pink-flowered plants. The anthocyanin content from F_1_ hybrid flowers was indistinguishable from wild-type plants (Fig. 3; Tukey's HSD for F1 vs. MPL, *P* = 0.965), suggesting that pink is dominant to white. In addition, only two discrete flower color classes segregated in the F_2_ that were similar in appearance to the pink and white parental forms (Figs. 2, 3). Segregation patterns in 96 F_2_ hybrids (75 pink: 21 white) did not differ significantly from the 3∶1 expectation that a single locus with complete dominance was responsible for flower color differences (G = 0.52, *P* = 0.50).

### *MlDfr* is a candidate gene for color variation

The absence of cyanidin from both the floral and vegetative tissue of the white-flowered plants suggests that the anthocyanin pathway is blocked in both tissues. As an initial screen to test if variation in flower color co-segregated with sequence variation in enzyme-coding anthocyanin genes, we sequenced partial coding regions at four of these loci (*Chs, F3h, Dfr*, and *Ans*) from 5 white-flowered and 5 pink-flowered F_2_s. Of the 39 SNPs identified among the genes, only *MlDfr* contained SNPs that co-segregated with flower color. Because of the Mendelian nature of this flower color transition, we focused our efforts further on the genomic region containing *MlDfr*.

We subsequently genotyped 96 F_2_ hybrids at a synonymous single-nucleotide difference in the coding sequence of *MlDfr*. Assuming a recessive loss-of-function mutation responsible for white flowers, allelic variation in *MlDfr* co-segregated perfectly with flower color. All F_1_ plants were pink-flowered and heterozygous. In the F_2_, all white-flowered individuals were homozygous for the *white* allele (i.e., that derived from the white-flowered parent in the cross), while pink-flowered plants were either heterozygous or homozygous for the wild-type, *pink* allele. Similar to patterns observed in the parental plants, white-flowered F_2_ plants did not produce any detectable cyanidin. Moreover, consistent with the hypothesis of a recessive loss-of-color mutation at *MlDfr*, cyanidin production did not vary between the heterozygous and homozygous pink-flowered F_2_ hybrids (ANOVA with all three F_2_ genotypes, F = 14.1, *P*<0.005; Tukey's HSD for heterozygous (absorbance mean [95%CI]: 0.836 [0.68, 0.99]) vs. homozygous (0.942 [0.50, 1.38]) pink-flowered F_2_, *P*>0.05).

While this pattern of co-segregation in an anthocyanin gene essential for pigment production is consistent with the hypothesis that a loss-of-function mutation in *MlDfr* is responsible for white flowers in the CRA population, additional hypotheses also must be considered. For example, it has been shown in closely related species (including *Mimulus aurantiacus* and *Antirrhinum majus*) that the *Dfr* gene is linked to anthocyanin-regulating transcription factors of the R2R3-MYB family that are responsible for changes in floral pigment intensity between populations [30], [31]. Moreover, in all other species that have been examined, multiple anthocyanin genes have been shown to be co-regulated by a common set of transcription factors that always include members of the R2R3-MYB family [20], [21], [28]. Thus, it is possible that allelic variation in *MlDfr* is tracking a mutation in a tightly linked regulator of gene expression that is the cause of white flowers. Alternatively, a *cis*-regulatory mutation in *MlDfr* may be responsible for the flower color change. Given the single-locus control of flower color differences in *M. lewisii*, if a linked transcription factor were responsible for the differences in flower color, then the expression of multiple enzyme-coding genes should be affected. If a *cis*-regulatory mutation in *MlDfr* caused white flowers, then we would expect to observe reduced expression only of *MlDfr* in white flowers.

**Figure 4:**
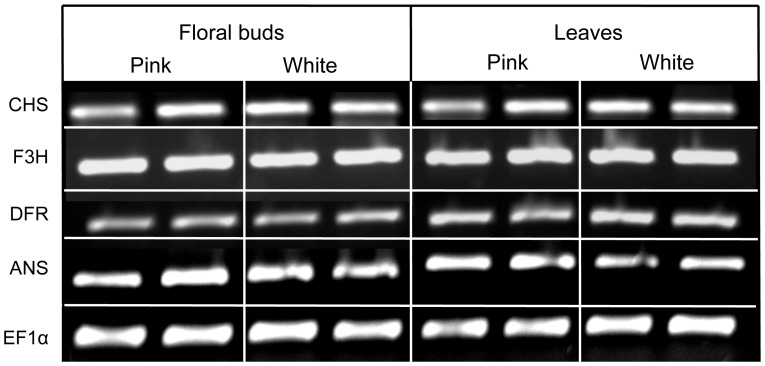
Qualitative expression levels of four core anthocyanin pathway enzymes. Shown are representative images of expression levels from floral and leaf tissues from two individuals of each morph. The genes are presented in the order they occur in the pathway. A constitutively expressed gene (*Ef1α*, coding for the translation elongation factor) was included in all experiments to control for difference in cDNA quality among samples.

Therefore, to determine if qualitative changes in gene expression were responsible for differences in flower color, we examined expression levels of the four pathway enzymes in different tissues from the two color morphs. Our assays using RT-PCR indicated that abundant transcript was produced from all four structural genes assayed in the vegetative and floral tissue of both the white-flowered and pink-flowered plants (Fig. 4). In addition, no substantial differences in *MlDfr* expression levels were detected between the color morphs or tissue types. While we cannot rule out minor changes in gene expression that also might contribute to the white-flowered phenotype, the complete loss of both floral and vegetative pigmentation in white-flowered plants is unlikely to be due exclusively to subtle differences in gene expression (either in *cis* or *trans*). Instead, these data suggest that a mutation (or mutations) in the coding sequence of *MlDfr* is primarily responsible for differences in pigment production between the color morphs.

### A frameshift mutation in *MlDfr* appears responsible for flower color differences

We cloned and sequenced the full-length *MlDfr* coding region from the white-flowered and the pink-flowered parents from our cross. The wild-type *MlDfr* allele contained six exons totaling 1392 bp, coding for a predicted protein of 464 amino acids. Nine amino acid differences were identified between the color morphs. In addition, a two-nucleotide (TG) insertion in the white-flowered plants occurred at position 265, causing a shift in the reading frame and a premature stop codon that terminates the protein at only about one-fourth of its functional length.

**Figure 5:**
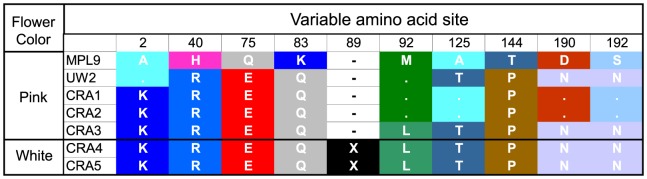
Amino acid positions that vary between the white-flowered CRA and pink-flowered MPL individuals in *MlDfr* . Sequences are included from white-flowered individuals from Crater Lake NP, Oregon (CRA4 and CRA5), and pink-flowered individuals from three populations (MPL9 – Modoc Plateau, Oregon; UW2 – Stevens Point, Washington; CRA1-3, Crater Lake NP, Oregon). Dots indicate conservation with the sequence from MPL9, and dashes indicate an alignment gap resulting from a 2-bp insertion in the white-flowered CRA sequences (represented by an X). The insertion is the only mutation unique to the two white-flowered CRA sequences.

The presence of this frameshift mutation suggests it is the likely causal mutation affecting flower color. However, because nine amino acid changes are also present between these sequences, it is possible that one (or several) of the non-synonymous mutations caused the loss of pigmentation in white flowers and the insertion arose subsequent to the initial loss-of-function mutation. To address this possibility, we sequenced the full-length *MlDfr* gene from an additional white-flowered plant from CRA and four additional pink-flowered individuals from two different populations, including accessions from Crater Lake. Of the nine amino acid changes, none were unique to white-flowered plants (Fig. 5), suggesting that they cannot be directly responsible for the loss of anthocyanin pigmentation in white-flowered CRA plants. By contrast, the only other change in this sequence is the 2-bp insertion. It is unique to these two white-flowered plants, which strongly suggests that it is the causal mutation responsible for white flowers, although it remains possible that it is instead in close linkage disequilibrium with the causal mutation.

### The *white* allele at *MlDfr* is rare in nature

To provide an unbiased estimate of the frequency of the *white MlDfr* allele in the natural population where it occurs, we genotyped the indel from 19 randomly selected plants from the field. By performing the collections after the plants ceased flowering, we were able to ensure that we did not preferentially sample rare white-flowered plants. Of these 19 plants, only one was heterozygous, and the remaining 18 individuals were homozygous for the *pink* allele. While white-flowered plants that are presumed to be homozygous for the insertion do occur rarely in this population, none were detected in our resampling of the population because of the random design of our collections. Based on 1000 bootstrap replicates in Arlequin v. 3.5 [33], we estimate the *white MlDfr* allele frequency to be approximately 0.026 (95% CI: [0, 0.079]).

## Discussion

Several lines of corroborating evidence suggest that white flowers in *M. lewisii* from the CRA population are the result of a frameshift mutation in exon two of *MlDfr*. Genetic crosses demonstrate that pink and white flowers segregate as a single locus, with pink dominant to white. Accordingly, genetic variation in *MlDfr* co-segregates perfectly with flower color in F_2_ hybrids. Moreover, by sequencing *pink* and *white* alleles at *MlDfr* from other regional *M. lewisii* populations, we have identified a two-nucleotide insertion in the second exon that is the only sequence variant found exclusively in white-flowered plants. This mutation causes a frameshift and a greatly truncated protein of 106 amino acids (relative to the wild-type of 464 amino acids) that is likely to prevent anthocyanin pigment synthesis. Consistent with a null, loss-of-function allele, we find no anthocyanins produced in the leaves or flowers of white-flowered plants. While transgenic or other functional experiments are necessary to confirm that this allele is causal, the fact that flower color differences segregate as a single gene, that there are no substantial expression differences at *MlDfr* between pink and white flowers, and that only one sequence change is found exclusively in white-flowered plants provides compelling evidence that this is the mutation directly responsible for white flowers.

Stern and Orgogozo [9] demonstrated that null coding mutations caused phenotypic differences within species more frequently than between species. Conversely, they also showed that *cis*-regulatory mutations caused phenotypic differences between species more frequently than within species. They explained these observations due to inherent differences in the way that selection affects coding and *cis*-regulatory mutations over these timescales. In the short-term, it may be possible for coding mutations to segregate even though they may have deleterious pleiotropic effects. However, in order to persist over the long term, only those advantageous mutations with the fewest negative pleiotropic effects are expected to be fixed. Consequently, their observations are consistent with the common wisdom that *cis*-regulatory mutations have fewer, negative pleiotropic effects relative to coding mutations.

Streisfeld and Rausher [10] explored whether certain types of mutations affecting flower color change were preferentially fixed by selection, but they did not address whether the spectrum of mutations that fixed differed from the spectrum of mutations that segregates within populations. By combining the results from Streisfeld and Rausher [10] with additional, recently published results (including the data from this study), we can now provide an initial appraisal of this prediction.

To determine whether certain types of mutations affecting flower color transitions were fixed preferentially, Streisfeld and Rausher [10] revealed that all documented examples of variation in the intensity of floral anthocyanin pigmentation fixed between populations or species involved either *cis*-regulatory or coding-sequence mutations in one class of anthocyanin-regulating transcription factor: the R2R3-MYBs [10]. Moreover, two additional examples published since then also involved mutations in R2R3-MYBs [30], [34]. This remarkable pattern of genetic convergence occurs even though the rate of spontaneous mutation causing changes in floral pigmentation does not appear to be biased toward transcription factor mutations in general, or R2R3-MYBs in particular [10]. Consequently, the discrepancy between the types of mutations that arise and those that become fixed occurs due to a significant fixation bias favoring R2R3-MYB mutations.

In contrast, there are now four examples of the genetic characterization of pigment intensity mutations segregating within populations. In the normally purple-flowered *Ipomoea purpurea*, two different white-flowered mutants are found in natural populations in the southeastern USA. In one case, white flowers at the *A*-locus are caused by an insertion in the coding region of the gene encoding the pathway enzyme CHS [35]. The second of these mutations is encoded at the *W*-locus and is due to a deletion in the coding region of an R2R3-MYB transcription factor (*IpMyb1*) [36]. In an arctic mustard (*Parrya nudicaulis*) with a purple-white polymorphism, white flowers appear to be caused by a *cis*-regulatory mutation in *Chs* [37]. Finally, our data from *M. lewisii* suggest that a frameshift in the second exon of *MlDfr* is responsible for white flowers in the Crater Lake population. Thus, while all documented mutations that fix between populations occur in R2R3-MYB genes, a broader spectrum of floral-pigment intensity mutations appears to segregate within populations. This suggests that of those mutations that arise and alter floral color within populations, only a small set of them becomes fixed between populations.

While genetic drift in small populations is capable of altering allele frequencies, these observations are also consistent with Stern and Orogogozo's [9] hypothesis that selection typically prevents those mutations with more deleterious pleiotropic effects from reaching fixation. Even though it is impossible to know whether the segregating white-flowered alleles discussed here will eventually fix or be eliminated from the populations, we can take advantage of our knowledge of the potential pleiotropic consequences of mutations in the genes and regulators of the anthocyanin pathway to explain the contrasting patterns observed at the alternate scales. For example, the pathway enzymes responsible for anthocyanin biosynthesis also are required to synthesize the flavonoids, a diverse group of secondary compounds with important physiological and ecological roles, including protection against ultraviolet radiation, drought, and herbivory [38], [39]. Likewise, the bHLH and WDR transcription factors that regulate the pathway enzymes have a broad expression domain and are responsible for regulating multiple developmental processes in different tissues, including changes in vacuolar pH, as well as the regulation of trichomes, root hairs, and seed coat mucilage production [20], [40], [41]. Thus, even if the production of white flowers provided some fitness advantage in these populations, coding mutations in these genes that alter flower color are unlikely to fix because they harbor extensive deleterious pleiotropic effects.

For example, in *Ipomoea purpurea*, the white-flowered allele at the *A*-locus (caused by a coding mutation in *Chs*) is never found at a frequency above 0.005 and has been demonstrated to contain negative pleiotropic effects on fitness that include reductions in floral production, heat tolerance, and fecundity [42]–[44]. This occurs even though selfing rates are higher in white-flowered plants, resulting in a significant transmission advantage that should allow the white-flowered allele to rise in frequency. Thus, the deleterious pleiotropic effects of the coding mutation in this system appear to prevent the fixation of the white-flowered allele. At present, we do not have information about the adaptive value of white flowers in *M. lewisii* or the potential pleiotropic consequences of the *MlDfr* mutation. However, based on the position of *Dfr* in the anthocyanin pathway, a loss of function mutation would prevent the synthesis of not only anthocyanin pigments, but also several flavonoid compounds that are produced downstream of DFR, including the proanthocyanidins. These compounds have been shown to be important in protecting plants from both microbial pathogens and insect herbivores [45]. Future experiments in this Crater Lake population are planned to look for pleiotropic effects of each allele that differentially affect fitness, which could help to explain why white flowers in this population are also rare.

In contrast to coding mutations in the pathway enzymes and most of their regulators, widespread gene duplication appears to have allowed individual copies of R2R3-MYBs to become tissue-specific, such that a single copy may regulate pigment intensity specifically in flowers while another copy regulates pigmentation in the vegetative tissues. Therefore, like *cis*-regulatory mutations, coding mutations in R2R3-MYBs are believed to harbor few negative, pleiotropic effects [46], which suggests that they will have a high probability of fixation. Thus, the expected reduction of negative pleiotropy associated with pigment-altering R2R3-MYB mutations may help to explain their elevated fixation rate between populations. Moreover, despite yielding a similar phenotype to the *A*-locus mutation in *I. purpurea*, the white-flowered allele at the *W*-locus in this same species (caused by a mutation in an R2R3-MYB) can occasionally reach frequencies of 0.5, with no detectable deleterious pleiotropic effects on fitness associated with this allele [47]. Thus, these data, while limited, further support our predictions about the role of pleiotropy among different types of flower color mutations.

In conclusion, we have shown that a frameshift mutation in an anthocyanin pathway enzyme is likely responsible for the rare, white-flowered polymorphism found in a population of *M. lewisii*. By comparing genetic data from other floral pigment intensity mutants segregating within populations with those that have fixed between populations, we have generated preliminary support for the hypothesis put forth by Stern and Orgogozo [9] that the genetic bases of short-term and long-term flower color evolution appear to differ. While mutations across much of the anthocyanin pathway and its regulatory network appear to be restricted from contributing to fixed differences in pigment intensity between populations, a broader set of phenotypically-similar mutations are capable of segregating - at least for a short while - within populations. These results suggest that even though the genetic basis of fixed differences in flower color may be predictable, segregating variation in flower color is not.

While we realize that our sample sizes are small and that patterns may change as more examples are characterized, our results are consistent with expected differences in the relative magnitude of deleterious pleiotropy among these mutation types. To confirm that differences in pleiotropy are responsible for these patterns, empirical studies are necessary to quantify the fitness effects associated with different types of mutations under field conditions. Moreover, to generate more robust support for these conclusions, additional examples are necessary where the genetic basis of both segregating and fixed differences in flower color are characterized. If these patterns continue to hold, as our results suggest they might, these analyses have the potential to contribute substantially to our understanding of the mechanisms that influence the predictability of the evolutionary process.

## Supporting Information

Table S1 **Degenerate primers for cloning, designed based on conserved regions in alignments from two closely-related** ***Mimulus*** **species.**
(DOCX)

Table S2 ***Mimulus lewisii*** **species–specific primers for qualitative PCR assays, designed from cloned sequences.**
(DOCX)

## References

[1] Hoekstra HE, Hirschmann RJ, Bundey RA, Insel PA, Crossland JP (2006) A single amino acid mutation contributes to adaptive beach mouse color pattern. Science 313: 101–104. 16825572 10.1126/science.1126121

[2] Rebeiz M, Pool JE, Kassner VA, Aquadro CF, Carroll SB (2009) Stepwise modification of a modular enhancer underlies adaptation in a *Drosophila* population. Science 326: 1663–1667. 20019281 10.1126/science.1178357 PMC3363996

[3] Wittkopp PJ, Stewart EE, Arnold LL, Neidert AH, Haerum BK, et al (2009) Intraspecific polymorphism to interspecific divergence: genetics of pigmentation in *Drosophila* . Science 326: 540–544. 19900891 10.1126/science.1176980

[4] Chan YF, Marks ME, Jones FC, Villarreal G, Shapiro MD, et al (2010) Adaptive evolution of pelvic reduction in sticklebacks by recurrent deletion of a *Pitx1* enhancer. Science 327: 302–305. 20007865 10.1126/science.1182213 PMC3109066

[5] Stern DL (2000) Perspective: Evolutionary developmental biology and the problem of variation. Evolution 54: 1079–1091. 11005278 10.1111/j.0014-3820.2000.tb00544.x

[6] Carroll SB (2005) Evolution at two levels: on genes and form. Plos Biology 3: e245. 16000021 10.1371/journal.pbio.0030245 PMC1174822

[7] Hoekstra HE, Coyne JA (2007) The locus of evolution: evo devo and the genetics of adaptation. Evolution 61: 995–1016. 17492956 10.1111/j.1558-5646.2007.00105.x

[8] Wray GA (2007) The evolutionary significance of *cis*-regulatory mutations. Nature Reviews Genetics 8: 206–216. 10.1038/nrg2063 17304246

[9] Stern DL, Orgogozo V (2008) The loci of evolution: How predictable is genetic evolution? Evolution 62: 2155–2177. 18616572 10.1111/j.1558-5646.2008.00450.x PMC2613234

[10] Streisfeld MA, Rausher MD (2011) Population genetics, pleiotropy, and the preferential fixation of mutations during adaptive evolution. Evolution 65: 629–642. 21054357 10.1111/j.1558-5646.2010.01165.x

[11] Conte GL, Arnegard ME, Peichel CL, Schluter D (2012) The probability of genetic parallelism and convergence in natural populations. Proceedings of the Royal Society B-Biological Sciences 279: 5039–5047. 10.1098/rspb.2012.2146 PMC3497250 23075840

[12] Stern DL, Orgogozo V (2009) Is genetic evolution predictable? Science 323: 746–751. 19197055 10.1126/science.1158997 PMC3184636

[13] Otto SP (2004) Two steps forward, one step back: the pleiotropic effects of favoured alleles. Proceedings of the Royal Society B-Biological Sciences 271: 705–714. 10.1098/rspb.2003.2635 PMC1691650 15209104

[14] Orr HA (2000) Adaptation and the cost of complexity. Evolution 54: 13–20. 10937178 10.1111/j.0014-3820.2000.tb00002.x

[15] Gompel N, Prud'homme B (2009) The causes of repeated genetic evolution. Developmental Biology 332: 36–47. 19433086 10.1016/j.ydbio.2009.04.040

[16] Nosil P, Harmon LJ, Seehausen O (2009) Ecological explanations for (incomplete) speciation. Trends in Ecology & Evolution 24: 145–156. 19185951 10.1016/j.tree.2008.10.011

[17] Rausher MD (2008) Evolutionary transitions in floral color. International Journal of Plant Sciences 169: 7–21.

[18] Warren J, Mackenzie S (2001) Why are all colour combinations not equally represented as flower-colour polymorphisms? New Phytologist 151: 237–241. 10.1046/j.1469-8137.2001.00159.x 33873384

[19] Holton TA, Cornish EC (1995) Genetics and biochemistry of anthocyanin biosynthesis. Plant Cell 7: 1071–1083. 12242398 10.1105/tpc.7.7.1071 PMC160913

[20] Koes R, Verweij W, Quattrocchio F (2005) Flavonoids: a colorful model for the regulation and evolution of biochemical pathways. Trends in Plant Science 10: 236–242. 15882656 10.1016/j.tplants.2005.03.002

[21] Quattrocchio F, Baudry A, Lepiniec L, Grotewold E (2006) The regulation of flavonoid biosynthesis. In: Grotewold E, editor. The Science of Flavonoids. New York: Springer. pp. 97–122.

[22] Hiesey WM, Nobs MA, Bjorkman O (1971) Experimental studies on the nature of species. V. Biosystematics, genetics, and physiological ecology of Erythranthe section of *Mimulus*. Washington, D. C.: Carnegie Institute.

[23] Hickman JC (1993) The Jepson Manual: Higher plants of California. Berkeley, CA: University of California Press.

[24] Schemske DW, Bradshaw HD (1999) Pollinator preference and the evolution of floral traits in monkeyflowers (*Mimulus*). Proceedings of the National Academy of Sciences of the United States of America 96: 11910–11915. 10518550 10.1073/pnas.96.21.11910 PMC18386

[25] Beardsley PM, Yen A, Olmstead RG (2003) AFLP phylogeny of *Mimulus* section Erythranthe and the evolution of hummingbird pollination. Evolution 57: 1397–1410. 12894947 10.1111/j.0014-3820.2003.tb00347.x

[26] Wilbert SM, Schemske DW, Bradshaw HD (1997) Floral anthocyanins from two monkeyflower species with different pollinators. Biochemical Systematics and Ecology 25: 437–443.

[27] Harborne JB (1984) Phytochemical Methods. New York: Chapman and Hall.

[28] Mol J, Grotewold E, Koes R (1998) How genes paint flowers and seeds. Trends in Plant Science 3: 212–217.

[29] Streisfeld M, Rausher M (2009) Altered *trans*-regulatory control of gene expression in multiple anthocyanin genes contributes to adaptive flower color evolution in *Mimulus aurantiacus* . Molecular Biology and Evolution 26: 433–444. 19029190 10.1093/molbev/msn268

[30] Streisfeld MA, Young WN, Sobel JM (2013) Divergent selection drives genetic differentiation in an R2R3-Myb transcription factor that contributes to incipient speciation in *Mimulus aurantiacus* . PloS Genetics 9: e1003385. 23555295 10.1371/journal.pgen.1003385 PMC3605050

[31] Schwinn K, Venail J, Shang YJ, Mackay S, Alm V, et al (2006) A small family of MYB-regulatory genes controls floral pigmentation intensity and patterning in the genus *Antirrhinum* . Plant Cell 18: 831–851. 16531495 10.1105/tpc.105.039255 PMC1425845

[32] Morita Y, Saitoh M, Hoshino A, Nitasaka E, Iida S (2006) Isolation of cDNAs for R2R3-MYB, bHLH and WDR transcriptional regulators and identification of *c* and *ca* mutations conferring white flowers in the Japanese morning glory. Plant Cell Physiol 47: 457–470. 16446312 10.1093/pcp/pcj012

[33] Excoffier L, Laval G, Schneider S (2005) Arlequin ver. 3.0: An integrated software package for population genetic data analysis. Evolutionary Bioinformatics Online 1: 47–50. PMC2658868 19325852

[34] Hopkins R, Rausher MD (2011) Identification of two genes causing reinforcement in the Texas wildflower *Phlox drummondii* . Nature 469: 411–415. 21217687 10.1038/nature09641

[35] Habu Y, Hisatomi Y, Lida S (1998) Molecular characterization of the mutable *flaked* allele for flower variegation in the common morning glory. Plant Journal 16: 371–376. 9881157 10.1046/j.1365-313x.1998.00308.x

[36] Chang SM, Lu YQ, Rausher MD (2005) Neutral evolution of the nonbinding region of the anthocyanin regulatory gene *Ipmyb1* in *Ipomoea* . Genetics 170: 1967–1978. 15944366 10.1534/genetics.104.034975 PMC1449781

[37] Dick CA, Buenrostro J, Butler T, Carlson ML, Kliebenstein DJ, et al (2011) Arctic mustard fower color polymorphism controlled by petal-specific downregulation at the threshold of the anthocyanin biosynthetic pathway. Plos One 6: e18230. 21490971 10.1371/journal.pone.0018230 PMC3072389

[38] Koes RE, Quattrocchio F, Mol JNM (1994) The flavonoid biosynthetic pathway in plants - function and evolution. Bioessays 16: 123–132.

[39] Winkel-Shirley B (2002) Biosynthesis of flavonoids and effects of stress. Current Opinion in Plant Biology 5: 218–223. 11960739 10.1016/s1369-5266(02)00256-x

[40] Broun P (2005) Transcriptional control of flavonoid biosynthesis: A complex network of conserved regulators involved in multiple aspects of differentiation in *Arabidopsis* . Current Opinion in Plant Biology 8: 272–279. 15860424 10.1016/j.pbi.2005.03.006

[41] Ramsay NA, Glover BJ (2005) MYB-bHLH-WD40 protein complex and the evolution of cellular diversity. Trends in Plant Science 10: 63–70. 15708343 10.1016/j.tplants.2004.12.011

[42] Coberly LC, Rausher MD (2003) Analysis of a chalcone synthase mutant in *Ipomoea purpurea* reveals a novel function for flavonoids: amelioration of heat stress. Molecular Ecology 12: 1113–1124. 12694276 10.1046/j.1365-294x.2003.01786.x

[43] Fehr C, Rausher MD (2004) Effects of variation at the flower-colour A locus on mating system parameters in *Ipomoea purpurea* . Molecular Ecology 13: 1839–1847. 15189207 10.1111/j.1365-294X.2004.02182.x

[44] Coberly LC, Rausher MD (2008) Pleiotropic effects of an allele producing white flowers in *Ipomoea purpurea* . Evolution 62: 1076–1085. 18298642 10.1111/j.1558-5646.2008.00355.x

[45] Dixon RA, Xie DY, Sharma SB (2005) Proanthocyanidins - a final frontier in flavonoid research? New Phytologist 165: 9–28. 15720617 10.1111/j.1469-8137.2004.01217.x

[46] Martin C, Ellis N, Rook F (2010) Do transcription factors play special roles in adaptive variation? Plant Physiol 154: 506–511. 20921174 10.1104/pp.110.161331 PMC2949032

[47] Rausher MD, Fry JD (1993) Effects of a locus affecting floral pigmentation in *Ipomoea purpurea* on female fitness components. Genetics 134: 1237–1247. 8375658 10.1093/genetics/134.4.1237 PMC1205591

