# Supplementary material for: The Genetic Basis of a Rare Flower Color Polymorphism in Mimulus lewisii Provides Insight into the Repeatability of Evolution
Source: PLoS One. 2013 Dec 3;8(12):e81173. doi: 10.1371/journal.pone.0081173 (PMC3849174; doi:10.1371/journal.pone.0081173)
Supplement: Table S1 — Degenerate primers for cloning, designed based on conserved regions in alignments from two closely-related Mimulus species. (DOCX) [file pone.0081173.s001.docx]

**Table S1.**  **Degenerate primers for cloning, designed based on conserved regions in alignments from two closely-related *Mimulus* species.**

| Locus | Sequence (5' - 3') |
| --- | --- |
| *Chs* | F: CGAGAAGTCGCAGATCAACA |
|  | R: GGATTTCCTCATTTCGTCCA |
| *F3h* | F: ATGTCTGGMGGGAAGAAAGG |
|  | R: TGAACCTCCCATTGCTTAGAT |
| *Dfr* | F: GGAAGCTACGATGAAGCAGTC |
|  | R: CTCTGCAAGTCTCGATGGCT |
| *Ans* | F: TGCCACGAKGAGGTGAAGAAGGC |
|  | R: CTCRGCCTCGGAAACCGTCTC |
