# Supplementary material for: The Genetic Basis of a Rare Flower Color Polymorphism in Mimulus lewisii Provides Insight into the Repeatability of Evolution
Source: PLoS One. 2013 Dec 3;8(12):e81173. doi: 10.1371/journal.pone.0081173 (PMC3849174; doi:10.1371/journal.pone.0081173)
Supplement: Table S2 — Mimulus lewisii species–specific primers for qualitative PCR assays, designed from cloned sequences. (DOCX) [file pone.0081173.s002.docx]

**Table S2. *Mimulus lewisii* species–specific primers for qualitative PCR assays, designed from cloned sequences.**

| Locus | Sequence (5' - 3') |
| --- | --- |
| *Chs* | F: CGAGAAGTCGCAGATCCACA |
|  | R: GGATTTCCTCATTTCGTCCA |
| *F3h* | F: CCGGGATTACTCGAGATGGC |
|  | R: AACGGGTTGAACCGTGATCC |
| *Dfr* | F: GCCAACAATTGAAGGTATGATG |
|  | R: CATCCACATGCACAAATTGC |
| *Ans* | F: CGTAGAACAGCTGCAGACCT |
|  | R: GGGATTTGTCCATTTGGCCC |
